# Prostate Health Index (Phi) and Prostate Cancer Antigen 3 (PCA3) Significantly Improve Prostate Cancer Detection at Initial Biopsy in a Total PSA Range of 2–10 ng/ml

**DOI:** 10.1371/journal.pone.0067687

**Published:** 2013-07-04

**Authors:** Matteo Ferro, Dario Bruzzese, Sisto Perdonà, Ada Marino, Claudia Mazzarella, Giuseppe Perruolo, Vittoria D’Esposito, Vincenzo Cosimato, Carlo Buonerba, Giuseppe Di Lorenzo, Gennaro Musi, Ottavio De Cobelli, Felix K. Chun, Daniela Terracciano

**Affiliations:** 1 Urology Unit, Fondazione G. Pascale, Napoli, Italy; 2 Department of Preventive Medical Sciences, University “Federico II”, Naples, Italy; 3 Urology Unit, University “Federico II”, Naples, Italy; 4 Department of Translational Medical Sciences, University “Federico II”, Naples, Italy; 5 Oncology Unit, University “Federico II”, Naples, Italy; 6 Division of Urology, European Institute of Oncology, Milan, Italy; 7 Department of Urology, University Medical Center Hamburg-Eppendorf, Hamburg, Germany; Innsbruck Medical University, Austria

## Abstract

Many efforts to reduce prostate specific antigen (PSA) overdiagnosis and overtreatment have been made. To this aim, Prostate Health Index (Phi) and Prostate Cancer Antigen 3 (PCA3) have been proposed as new more specific biomarkers. We evaluated the ability of phi and PCA3 to identify prostate cancer (PCa) at initial prostate biopsy in men with total PSA range of 2–10 ng/ml. The performance of phi and PCA3 were evaluated in 300 patients undergoing first prostate biopsy. ROC curve analyses tested the accuracy (AUC) of phi and PCA3 in predicting PCa. Decision curve analyses (DCA) were used to compare the clinical benefit of the two biomarkers. We found that the AUC value of phi (0.77) was comparable to those of %p2PSA (0.76) and PCA3 (0.73) with no significant differences in pairwise comparison (%p2PSA vs phi p = 0.673, %p2PSA vs. PCA3 p = 0.417 and phi vs. PCA3 p = 0.247). These three biomarkers significantly outperformed fPSA (AUC = 0.60), % fPSA (AUC = 0.62) and p2PSA (AUC = 0.63). At DCA, phi and PCA3 exhibited a very close net benefit profile until the threshold probability of 25%, then phi index showed higher net benefit than PCA3. Multivariable analysis showed that the addition of phi and PCA3 to the base multivariable model (age, PSA, %fPSA, DRE, prostate volume) increased predictive accuracy, whereas no model improved single biomarker performance. Finally we showed that subjects with active surveillance (AS) compatible cancer had significantly lower phi and PCA3 values (p<0.001 and p = 0.01, respectively). In conclusion, both phi and PCA3 comparably increase the accuracy in predicting the presence of PCa in total PSA range 2–10 ng/ml at initial biopsy, outperforming currently used %fPSA.

## Introduction

The widespread use of PSA screening and extended prostate biopsy protocols strongly increased the incidence of PCa and the detection of low-risk tumors that may not clinically progress during lifetime. However, preoperative tools (such as PSA and DRE) lack accuracy to avoid many negative biopsies and to predict confined PCa at radical prostatectomy (RP) [1].

Thus, several studies investigated the ability of new biomarkers to improve PCa diagnosis reducing unnecessary biopsies and to discriminate between aggressive and slow-growing cancers avoiding overtreatment. Recently, Prostate cancer antigen 3 (PCA3) and phi (prostate health index) have been proposed as useful tools in prostate cancer patient care [2]–[8].

In 2012 PCA3 was approved by the US Food and Drug Administration (FDA) for the use in men scheduled for repeat biopsy and [−2]proPSA for initial biopsy decisions in men with PSA concentrations in the range of 4–10 ng/ml and negative DRE.

Recently, Stephan et al [9] compared urinary PCA3, transmembrane protease, serine 2 (*TMPRSS2*):v-ets erythroblastosis virus E26 oncogene homolog (avian) (*ERG*) gene fusion (T2:ERG), and the serum phi for predicting biopsy outcome in a multicentre study including men with PSA values between 0–20 ng/ml undergoing first and repeat biopsy.

In addition, Scattoni et al [10] in a two centers study reported that phi performed better than PCA3 as predictor of outcome both in the initial and repeated biopsy.

Still, a direct comparison of phi and PCA3 in a single centre study in subjects undergoing first biopsy with PSA values comprised in the “grey” zone 2–10 ng/ml has not been available until now.

Therefore, the aim of the present study was to compare the diagnostic ability of PCA3 and phi in men who had undergone initial biopsies. Moreover, we stratified patient risk before treatment, according to PRIAS criteria [11], thus we evaluated not only the ability of the two biomarkers to detect PCa, but also their correlation with active surveillance (AS) elegibility.

## Materials and Methods

### Study Population

Before prostate biopsy (minimum 16 cores), 332 subjects were enrolled in a prospective observational study, approved by the hospital ethics committee. Blood and urine specimens were collected according to predetermined standard operating procedure [12]. Participants provided written approved consent. Ethical approval for this study was given by the institutional Ethics Committee of the IRCCS Fondazione G. Pascale, Napoli, Italy (M2/33).

Among these, 300 met eligibility criteria for this study: age over 50 years, no prior prostate surgery and biopsy, no bacterial acute or chronic prostatitis, no use of 5-α reductase inhibitors, PSA values included between 2 and 10 ng/ml, availability of serum samples and corresponding clinical data and completion of at least a 16 core template biopsy after enrollment. The final study cohort included 108 PCa patients (36%) and 192 (64%) with no evidence of malignancy (NEM).

### Methods

Participants had blood drawn before DRE at each visit. Whole blood was allowed to clot before serum was separated by centrifugation. Serum aliquots were stored at −80°C until samples were processed, according to Semjonow et al [13]. Specimens were analyzed in blinded fashion for PSA, fPSA and p2PSA by Access2 Immunoassay System analyzer (Beckman Coulter, Brea, CA, USA) calibrated against the WHO standard for PSA and fPSA. The analytical performance of the measurements assessed with control materials (Beckman Coulter) showed values within the allowed recommended limits.

After a DRE with 3 strokes per lobe as described earlier [14], urine samples were collected (PROGENSA PCA3 urine sample collection kit, Gen-Probe), and the PROGENSA PCA3 assay (Gen-Probe, San Diego, CA, USA) was performed retrospectively in all samples.

The PCA3 score was calculated as: (mRNA PCA3)/(mRNA PSA) X 1000. Transrectal ultrasonography was used to determine prostate volume.

Patients underwent prostate biopsies according to a standardized institutional saturation scheme, which consisted of at least 16 needle biopsy cores obtained under transrectal-ultrasound (TRUS) guidance. Primary and secondary Gleason score were assigned by a single genitourinary pathologist blinded to the biomarkers values, according to the 2005 consensus conference of the International Society of Urological Pathology definitions [15].

### Statistical Analysis

All statistical analysis were performed in R (R Development Core Team, 2012).

Median [min**–**max] were used to describe continuous variables while categorical variables were reported as number of occurrences and percentages. The Mann-Whytney and Chi-square test were used to asses differences among PCa and NEM subjects. The predictive accuracy of the single markers was measured by the Area under the ROC curve (AUC). Differences in diagnostic performance were assessed using the De Long method. Because of the large number of the pairwise comparisons among markers and to control the family-wise error rate at level α = 0.05, the significance of the DeLong test statistics was appraised by using the adaptive Bonferroni procedure [16]. Sensitivities and specificities at different cutoff (high sensitivity and best combination of sensitivity and specificity) were compared using the Mc Nemar test for paired proportions. Multivariable logistic regression analysis was performed to assess whether addition of either PCA3 or phi increased the predictive accuracy of a base set of predictors routinely used as screening tool for the detection of prostate cancer. Finally, Decision Curve analysis [17] was used to compare the net benefit (calculated by subtracting the proportions of false positive from the proportion of true positive, the former being weighted by the relative harms of false positive and false negative results) of using phi and PCA3 in guiding initial biopsy decision. Statistical significance was set at p<0.05 (unless in AUC pairwise comparisons as above stated).

## Results

The demographic and clinical characteristics of the study population (n = 300) are listed in Table 1.

**Table 1:** Clinical characteristics of study population.

	PCa	NEM	Overall Population		
	(n = 108)	(n = 192)	(n = 300)	p	
Age, media	65	58	60	<0.001	†
(min-max), years	(50–73)	(50–70)	(50–73)		
tPSA, median	6.42	6.11	6.18	0.58	†
(min-max), ng/ml	(2.11–10)	(2.12–10)	(2.11–10)		
fPSA, median	0.87	1.09	0.97	0.003	†
(min-max), ng/ml	(0–4)	(0–4)	(0–4)		
p2PSA, median	20.08	16.08	17.04	<0.001	†
(min-max),pg/ml	(3.75–99.12)	(3.54–102.82)	(3.54–102.82)		
phi, median	52.45	35.88	41.04	<0.001	†
(min-max)	(14.37–210.62)	(14.02–109.92)	(14.02–210.62)		
PCA3, median	60	34	40	<0.001	†
(min-max)	(10–250)	(2–257)	(2–257)		
%fPSA, median	0.17	0.19	0.19	0.001	†
(min-max)	(0.03–0.60)	(0.05–0.70)	(0.03–0.7)		
%p2PSA,median	2.31	1.5	1.76	<0.001	†
(min-max)	(0.51–10.84)	(0.51–4.2)	(0.51–10.84)		
Prostate volume,	50	50	50	0.413	†
median(min-max),cc	(20–90)	(25–130)	(20–130)		
PSA density,median	0.12	0.11	0.11	0.266	†
(min-max)	(0.05–0.26)	(0.03–0.30)	(0.030.30)		
DRE positive, n (%)	36 (33)	22 (11)	58 (19)	<0.001	††
Family History positive, n (%)	14 (13)	8 (4)	22 (7)	0.010	††

† Mann Whitney test.

†† Chi-square test.

The mean age (± SD) of all the subjects included in this study was 60.4±6.0 years. Positive biopsy for PCa was found in 108 (36%) patients. No significant differences were found between patients with NEM and those with PCa for PSA, prostate volume and PSA density, (p = 0.580, 0.413 and 0.266 respectively). Age (p<0.001), fPSA (p 0.003), %fPSA (p 0.001), p2PSA (<0.001), %p2PSA (<0.001), phi (<0.001), PCA3 (<0.001), percentage of positive DRE (p<0.001) and positive family history (p = 0.01) differed significantly between PCa and NEM patients.

### Univariable Analysis

**Figure 1:**
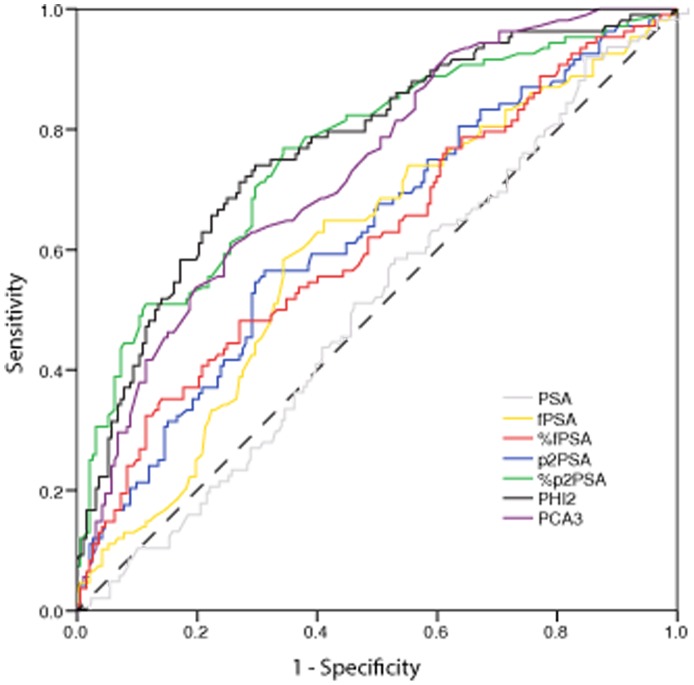
Receiver operating characteristic (ROC) curve for comparing all the analyzed markers as predictor of PCa in first biopsy.

In table 2 and in figure 1, the results of ROC curve analysis for all the markers are shown. The largest AUC’s were obtained with phi (0.77; 95% C.I. 0.72 to 0.83), %p2PSA (0.76 with 95% C.I. 0.71 to 0.82) and PCA3 (0.73 with 95% C.I. 0.68 to 0.79) with no significant differences in pairwise comparison (p = 0.673 comparing %p2PSA vs phi, p = 0.417 comparing %p2PSA vs PCA3 and p = 0.247 comparing phi vs PCA3). All of them outperformed fPSA and %fPSA even after Bonferroni correction for multiple comparisons (p<0.05 DeLong method; data not shown). Phi, %p2PSA and PCA3 also showed comparable levels of specificity at high level (90%) of sensitivity (p>0.05, Mac-Nemar test; data not shown). When considering the cutoff achieving the best combination of sensitivity and specificity, both %p2PSA and phi showed a significant improvement in sensitivity with respect to PCA3 (0.14, 95% C.I. [0.04 to 0.24], p = 0.01 and 0.19, 95% C.I. [0.08 to 0.30], p<0.01 respectively) without a significant difference in specificity.

**Table 2:** Results of the ROC curve analysis for all the studied markers.

	AUC (95% C.I.)	90% Sensitivity	
		Cut-off	Specificities (95% C.I.)
tPSA	0.52 (0.45 to 0.59)	3.6	0.17 (0.07 to 0.24)
fPSA	0.60 (0.54 to 0.67)	0.6	0.15 (0.06 to 0.29)
%fPSA	0.62 (0.55 to 0.69)	0.1	0.20 (0.12 to 0.28)
p2PSA	0.63 (0.56 to 0.69)	9.5	0.18 (0.10 to 0.31)
%p2PSA	0.76 (0.71 to 0.82)	1.3	0.36 (0.17 to 0.52)
phi	0.77 (0.72 to 0.83)	31.6	0.40 (0.27 to 0.52)
PCA3	0.73 (0.68 to 0.79)	22.0	0.40 (0.28 to 0.48)

### Multivariable Analysis

Both phi and PCA3 acted as independent predictors of PCa when added to a base set of predictors including age, PSA, % fPSA, EDR and prostate volume (Table 3). The diagnostic accuracy of the base multivariable model was significantly improved by the presence of phi (0.72, vs 0.82, p<0.001), PCA3 (0.72 vs 0.77 p = 0.015) and the combination of both markers (0.72 vs 0.83 p<0.001). However, the multivariable models did not improve the AUC over both phi and PCA3 as shown by univariable analysis (p>0.05, De Long method, data not shown).

**Table 3:** Predictive accuracy of the combined models in predicting the presence of PCa at initial biopsy.

	Base Model		Base Model+phi		Base Model+PCA3		Base Model+phi+PCA3	
Predictors of Pca	O.R. (95% C.I.)	*p*	O.R. (95% C.I.)	*p*	O.R. (95% C.I.)	*p*	O.R. (95% C.I.)	*p*
Age	1.10 (1.05 to 1.14)	<0.001	1.09 (1.04 to 1.14)	<0.001	1.08 (1.03 to 1.13)	<0.001	1.08 (1.03 to 1.14)	<0.001
PSA	0.97 (0.85 to 1.09)	0.575	0.84 (0.73 to 0.98)	0.023	0.94 (0.83 to 1.08)	0.382	0.85 (0.73 to 0.98)	0.027
% fPSA	0.02 (0.00 to 0.59)	0.024	0.08 (0.00 to 2.80)	0.167	0.04 (0.00 to 1.32)	0.071	0.10 (0.00 to 3.42)	0.198
DRE (positive vs negative)	3.47 (1.82 to 6.58)	<0.001	2.57 (1.26 to 5.24)	0.010	2.89 (1.48 to 5.67)	0.002	2.37 (1.14 to 4.93)	0.021
Prostate volume	0.99 (0.97 to 1.01)	0.215	0.99 (0.97 to 1.01)	0.507	0.99 (0.97 to 1.01)	0.279	0.99 (0.97 to 1.02)	0.547
phi	–	–	1.05 (1.03 to 1.07)	<0.001	–	–	1.05 (1.03 to 1.07)	<0.001
PCA3	–	–	–	–	1.02 (1.01 to 1.02)	<0.001	1.01 (1.00 to 1.02)	0.016
AUC (95% C.I.)	0.72 (0.67 to 0.79)		0.82 (0.77 to 0.87)		0.77 (0.72 to 0.83)		0.83 (0.78 to 0.87)	
*p°*	–		<0.001		0.025		<0.001	

°Compared with the base model - De Long method.

### DCA Analysis

**Figure 2:**
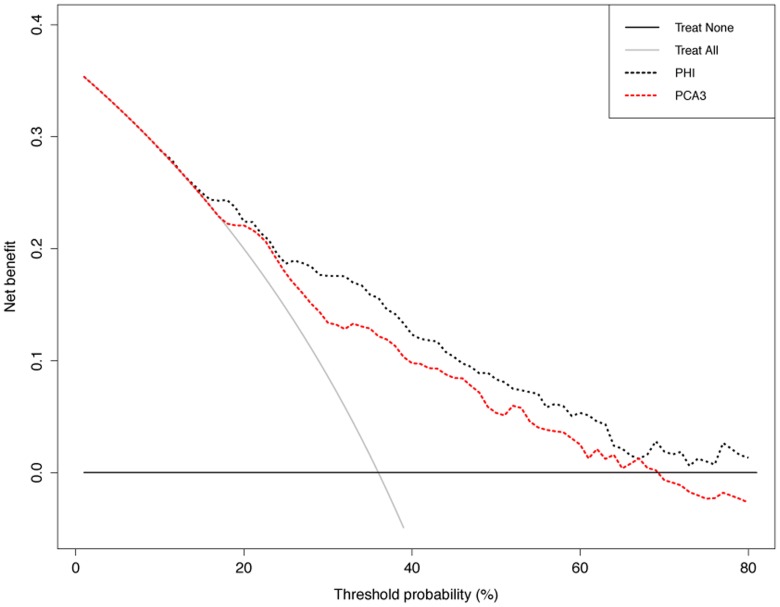
Decision curve analysis. The net benefit (calculated by subtracting the proportions of false positive from the proportion of true positive, the former being weighted by the relative harms of false positive and false negative results) of both phi and PCA3 is plotted against the threshold probability (the probability of PCa at which the benefits of opting for biopsy or no biopsy are considered equal). Solid lines represent the net benefit associated to the benchmarking strategies of biopsying all or no men irrespective of any diagnostic tool.

Results of the DCA analysis are reported in figure 2. Phi and PCA3 exhibited a very close net benefit profile until the threshold probability of 25% after that phi index had an increased net benefit against the PCA3 which endures for the defined range of clinically plausible threshold probabilities (10–40%) [18]. For example, at a threshold probability of 30%, the net benefit associated to phi index and PCA3 were equal to 17.6% and 13.4%, respectively. This means that using phi index as diagnostic tool led to a 21% reduction in the number of unnecessary biopsies without increasing the proportion of PCa who properly underwent to biopsy, compared to the reduction of 11% achieved by using PCA3.

### Univariable Analysis and Active Surveillance Predictors

**Figure 3:**
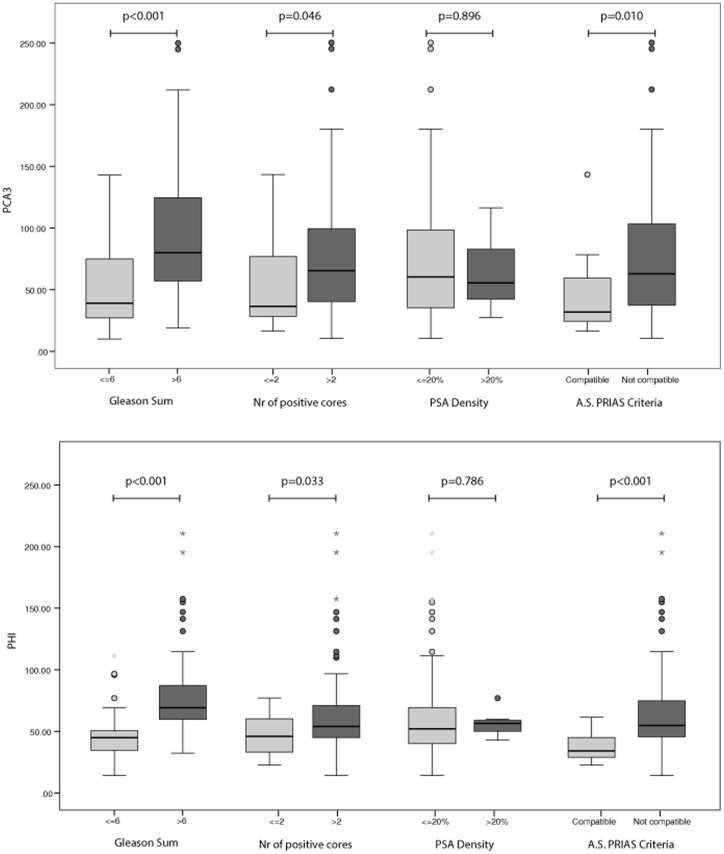
PCA3 and phi ability in discriminating PCa according to PRIAS criteria. Box plot shows the distribution of PCA3 values (upper panel) and phi values (lower panel) in patients with biopsy proven PCa classified according to the PRIAS criteria for active surveillance. Data are shown as median (horizontal line in the box) and Q1 and Q3 (borders of the box). Whiskers represent the lowest and the highest values that are not outliers (i.e., data points below Q1–1.5x IQR or above Q3+1.5x IQR) or extreme values (i.e., data points below Q1–3xIQR or above Q3+3xIQR). Dots represent outlier values and asterisks represent extreme values. Q1 = 25th percentile; Q3 = 75th percentile; IQR (interquartile range) = Q3–Q1.

In figure 3 we reported the results of univariable analysis testing the ability of either phi or PCA3 in discriminating favorable from unfavorable PCa according to PRIAS criteria (Biopsy Gleason score ≤6, number of positive cores ≤2 and PSA density ≤20%,). Individuals with prostate cancer compatible with AS had significantly lower phi and PCA3 values than those who did not match PRIAS criteria (p<0.001 and p = 0.01, respectively). Both phi and PCA3 exhibited a significant different profile between groups as regard Gleason score and number of positive cores, whereas none of the two biomarkers were associated to PSA density dichotomized at the value of 20%.

## Discussion

It is generally accepted that PSA leaves much to be desired as first-line diagnostic test and as predictive prognostic indicator. To supplement the information from PSA analysis several biomarkers have been proposed such as phi index and PCA3 score [2]–[7].

We recently reported [8] that PCA3 and phi perform comparably in 160 men undergoing first prostate biopsy with PSA values between 0 and 20 ng/ml. Accordingly, Stephan et al [9] found that the ability to detect PCa of PCA3 and phi were not significantly different in a multicentric cohort of 246 men undergoing first and repeat biopsies with PSA values between 0 and 20 ng/ml. In the same report the authors showed that although PCA3 had the largest AUC in the repeat biopsy cohort, in the 2–10 ng/ml tPSA range group with negative DRE and in the initial biopsy cohort the two biomarkers are similarly able to detect PCa. On the other hand, univariable and multivariable analysis performed by Scattoni et al [10] showed that phi was slightly more accurate than PCA3 both in first and repeated biopsy setting.

In this study we found that PCA3 and phi performed comparably in a study population including 300 subjects undergoing first biopsy with PSA values included in the “grey” zone 2–10 ng/ml. Differently from two previous studies [8], [9], the combination of the two biomarkers provided no additional enhancement of the diagnostic power in our cohort. This result may be explained on the basis of the study population selected by different inclusion criteria particularly DRE, family history and PSA values range used in the present study.

Multivariable analysis showed that the addition of phi and PCA3 to a base model including currently used PCa predictors significantly increases predictive accuracy, according to Scattoni et al [10]. However, the usefulness of multivariable models needs to be further and extensively investigated, because no model improved the performance of the single biomarker, as already reported in our previous study [5].

Our findings open discussion about whether phi or PCA3 could be recommended as the best single parameter in addition to PSA “grey” values as first line diagnostic test for PCa detection.

Recently [7], [19], two different prospective multicenter studies suggested that phi and %p2PSA provided significantly better clinical performance than other PSA molecular forms assays in detecting PCa in 2–10 ng/ml tPSA range. Hansen et al [20] demonstrated that PCA3 achieved independent predictor status of PCa in subjects undergoing first prostate biopsy. In studies comparing phi and PCA3 performance in mixed biopsy patient cohort [19], PCA3 score was more accurate than phi in the repeat biopsy setting.

Both biomarkers are superior to the treat all strategy of biopsying every patient only in a part (20–40%) of the defined range of interest for prostate cancer threshold probability interval [18]. Moreover, phi definitely surpasses PCA3 for threshold probabilities above 25%.

Therefore, owing to its easier and cheaper technology, its lower discomfort for the patients and its better ability to reduce unnecessary biopsies (as shown by DCA), phi should be probably recommended as the best assay in addition to PSA as first line diagnostic test for PCa detection.

Taken together, literature data suggested that PCA3 could be reserved for those patients undergoing repeat biopsies, for whom is a well-established biomarker [2]. On the other hand, phi emerges as a cheaper assay than PCA3, that hold promise to help urologist to plan to take or not first biopsy, especially if future investigations will further confirm phi test stability and reproducibility [7].

Moreover, in this study we firstly assessed the correlation of phi and PCA3 with prognostic biopsy outcome such as Gleason sum and positive core numbers and with PSA density. We found that the two biomarkers significantly correlate with Gleason sum higher than 6 and number of positive cores higher than 2 and inversely with AS criteria compatibility. Previously published reports investigated the value of phi or PCA3 to predict biopsy reclassification during AS[21]–[23] and pathological features at radical prostatectomy [24], [25]. Our encouraging results may help to improve the selection of patients eligible for active surveillance according to PRIAS criteria or for neurovascular bundle-sparing surgery. Future multinstitutional studies on larger population are needed to verify if combination of phi and PCA3 may improve biopsy reclassification in subjects enrolled in an AS program.

The strengthen of our study resides in a single centre dataset including subjects at first biopsy allowing us to assess the net clinical benefit of one marker over the other and to define cut-offs calculated on a large population. Unfortunately, PCa patients number is not enough to evaluate the ability of phi and PCA3 alone or in combination to predict clinically localized cancer compatible with watchful waiting.

### Conclusions

In patients with a tPSA between 2 and 10 ng/ml, phi and PCA3 are the strongest predictors of PCa and are significantly more accurate than the currently used tests in PCa detection. Moreover, the two biomarkers are strongly correlated with biopsy outcomes, suggesting a potential role in AS patients selection.
